# Supplementary material for: Experimentally determined traits shape bacterial community composition one and five years following wildfire
Source: Nat Ecol Evol. 2023 Jul 31;7(9):1419–31. doi: 10.1038/s41559-023-02135-4 (PMC10482699; doi:10.1038/s41559-023-02135-4)
Supplement: Supplementary file 1 — Supplementary Fig. 1, Tables 1–10 and 12–15, Notes and References. [file 41559_2023_2135_MOESM1_ESM.pdf]

nature ecology & evolution

Article https://doi.org/10.1038/s41559-023-02135-4

Experimentally determined traits shape bacterial community composition one and five years following wildfire

In the format provided by the authors and unedited

Table of Contents Page

Supplemental Figures

Supplementary Figure 1. Bray-Curtis dissimilarities between burned and unburned *vs.* change in pH 2

Supplemental Tables

Supplementary Table 1. Site characteristics, location, and soil properties 3
Supplementary Table 2. Mean recorded maximum soil temperatures during burns 4
Supplementary Table 3. Mean soil pH, total C, total N, and horizon thickness 5
Supplementary Table 4. Mean 2-pool decay coefficients for fast-cycling C pool 6
Supplementary Table 5. Mean 2-pool decay coefficients for slow-cycling C pool 6
Supplementary Table 6. PERMANOVA results for full model for 16S for cDNA 7
Supplementary Table 7. PERMANOVA results for full model for 16S genomic DNA 7
Supplementary Table 8. PERMANOVA results for full model at end of fast-growth incubation 8
Supplementary Table 9. PERMANOVA results for full model at end of post-fire affinity incubation 8
Supplementary Table 10. Observed OTUs in each laboratory sample 9
Supplementary Table 11. List of all OTUs identified for each fire-adaptive strategy 9
Supplementary Table 12. Fire-responding taxa by Phylum 9
Supplementary Table 13. Fire-responding taxa by Class 10
Supplementary Table 14. Mean fractional dry mass loss 10
Supplementary Table 15. Primers used 11
Supplementary Table 16. Full PCR primers with barcodes 11

Supplemental Notes 12

References 15

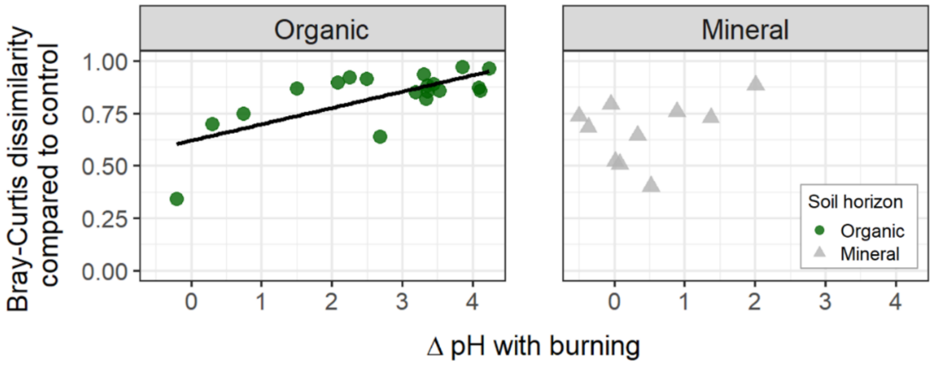

Supplementary Figure 1. Bray-Curtis dissimilarities in dry soil burn cores compared to unburned controls following autoclaving, inoculation with unburned soil, and a 6-month incubation in organic (left) and mineral (right) horizons *vs.* change in pH with burning. Line indicates linear model fit (y = 0.6+0.08x; $R^2_{adj.}$ = 0.50; P = 0.0008).

Supplementary Table 1. Site characteristics, location, and soil properties grouped by dominant vegetation type.

Site	Slope position	Aspect	Overstory density (%)	Latitude	Longitude	Sand (%)	Silt (%)	Clay (%)
***Picea* spp.**								
4	Middle	ESE	98	59° 27' 44.62" N	112° 17' 51.10" W	62.5	32.5	5
6	Lower	NW	52	59° 28' 19.14" N	112° 16' 23.58" W	70.5	19.5	10
7	Level	NNW	96	59° 35' 29.05" N	112° 16' 13.72" W	NA	NA	NA
12	Toe		91	59° 48' 28.97" N	112° 00' 18.41" W	NA	NA	NA
13	Level		79	59° 58' 24.99" N	112° 26' 57.74" W	NA	NA	NA
17	Level		92	60° 01' 48.23" N	112° 53' 21.29" W	NA	NA	NA
18	Level		92	60° 00' 54.13" N	112° 52' 42.88" W	33	37	30
Populus tremuloides								
2	Level		94	59° 27' 07.18" N	112° 19' 35.98" W	54.5	32	13.5
5	Middle	NW	97	59° 27' 41.18" N	112° 17' 00.31" W	59.5	31	9.5
8	Level	S	99	59° 38' 59.28" N	112° 12' 45.89" W	68	25	7
11	Level		99	59° 47' 45.65" N	112° 02' 27.45" W	59	32	9
14	Level		89	60° 02' 54.21" N	112° 47' 50.94" W	72	20	8
15	Lower	NW	93	60° 02' 21.03" N	112° 52' 45.09" W	70	19	11
Pinus banksiana								
1	Upper	W	96	59° 24' 38.19" N	112° 23' 50.15" W	23	43	34
3	Level		100	59° 27' 25.16" N	112° 19' 27.27" W	51.5	36	12.5
9	Level		97	59° 41' 04.03" N	112° 10' 18.51" W	54	29.5	16.5
10	Level		100	59° 42' 04.70" N	112° 10' 13.50" W	49	41	10
16	Level		85	60° 01' 17.15" N	112° 58' 14.23" W	50	32.5	17.5
19	Middle	SSE	91	60° 02' 05.49" N	113° 08' 40.44" W	93	3	4

Supplementary Table 2. Mean recorded maximum soil temperature and degree hours above 21 °C at the O horizon-mineral soil interface and 1 cm above core base. Standard deviation in parentheses.

Burn treatment	Soil horizon	Maximum temp. at O/Mineral interface (°C)	Maximum temp. 1 cm above base of core (°C)	Degree hours at O/Mineral interface	Degree hours 1 cm above base of core
***Picea* spp.**					
Dry soil	O (n=7)	357.3 (234.1)	152 (179)	699.3 (545.3)	242.2 (297.5)
	Mineral (n=3)	292.3 (249.1)	75.9 (68.2)	521 (606.4)	135.2 (163.8)
Moist soil	O (n=7)	29.9 (6.3)	28.6 (3.3)	19.3 (7.2)	26.3 (7.6)
	Mineral (n=2)	28.1 (2.7)	29 (1.7)	25.8 (0)	34.9 (5.4)
Pinus banksiana					
Dry soil	O (n=6)	359.6 (157.6)	71.5 (35.1)	487.5 (314.4)	108.4 (53.3)
	Mineral (n=6)	359.6 (157.6)	71.5 (35.1)	487.5 (314.4)	108.4 (53.3)
Moist soil	O (n=6)	30.5 (5.3)	26.8 (1.9)	15 (5.5)	19.2 (7.8)
	Mineral (n=6)	30.5 (5.3)	26.8 (1.9)	15 (5.5)	19.2 (7.8)
Populus tremuloides					
Dry soil	O (n=6)	439.5 (159)	199.6 (210.4)	745.9 (396.5)	323.5 (334.8)
	Mineral (n=4)	413.8 (195.9)	176.1 (230.5)	584.1 (309.3)	224.1 (246.5)
Moist soil	O (n=6)	28.1 (3.4)	25.9 (0.4)	16.7 (4.8)	20.2 (7)
	Mineral (n=4)	28 (4.1)	25.8 (0.5)	14.3 (3.9)	18.5 (7.5)

Supplementary Table 3. Mean soil pH, total C, total N, and horizon thickness for dry and moist soil burn samples and unburned soil grouped by dominant vegetation. Standard deviation in parentheses.

Burn treatment	Soil horizon	C:N	Total C (%)	Total N (%)	pH	Pre-burn horizon thickness (cm)	Post-burn horizon thickness (cm)
***Picea* spp.**							
Control	O (n=7)	22.6 (6.1)	37.8 (9.3)	1.7 (0.5)	4.5 (1)	8.9 (1.8)	8.9 (1.8)
	Mineral (n=2)	14.8 (5.4)	1.5 (0)	0.1 (0)	3.9 (0)	3.7 (1)	3.7 (1)
Dry soil	O (n=7)	14.9 (5.1)	20.5 (13.5)	1.3 (0.9)	6.6 (1.4)	8 (2.6)	5.5 (2.2)
	Mineral (n=3)	13.4 (6.3)	1.8 (1.1)	0.1 (0)	5.1 (1.2)	4.5 (1.8)	4.5 (1.8)
Moist soil	O (n=7)	21.8 (4.8)	35.2 (11.5)	1.6 (0.5)	5.1 (1)	9.2 (1.6)	8.1 (1.7)
	Mineral (n=2)	15.1 (4.6)	1.6 (0.1)	0.1 (0)	3.7 (0.3)	2.7 (2.4)	2.7 (2.4)
Pinus banksiana							
Control	O (n=6)	24 (4)	23.1 (9.6)	0.9 (0.5)	4.1 (0.4)	5.4 (0.5)	5.4 (0.5)
	Mineral (n=6)	16.3 (4.7)	0.8 (0.2)	0	4.1 (0.7)	4.6 (0.5)	4.6 (0.5)
Dry soil	O (n=6)	12.5 (3.9)	12.2 (9.8)	0.9 (0.6)	7.4 (0.3)	5.5 (0.8)	3.5 (0.8)
	Mineral (n=6)	14.4 (2.6)	1.2 (0.3)	0	4.6 (0.5)	4.5 (0.8)	4.5 (0.8)
Moist soil	O (n=6)	22.7 (0.5)	29.8 (7.7)	1.3 (0.3)	4.3 (0.6)	4.7 (0.8)	3.5 (0.9)
	Mineral (n=6)	15.7 (3.7)	1.2 (0.3)	0	4.1 (0.7)	5.2 (0.8)	5.2 (0.8)
Populus tremuloides							
Control	O (n=6)	14.8 (2.1)	19.6 (9.7)	1.3 (0.8)	4.7 (0.3)	7.3 (3)	7.3 (3)
	Mineral (n=4)	12.5 (2.4)	1.3 (0.3)	0.1 (0)	4.7 (1.1)	5.3 (1.1)	5.3 (1.1)
Dry soil	O (n=6)	10 (1.9)	13.7 (9.3)	1.4 (1)	7.5 (0.5)	7.5 (2.5)	5.3 (2)
	Mineral (n=4)	9 (3.7)	3.1 (2.5)	0.5 (0.6)	4.7 (1.1)	3.5 (2.6)	3.5 (2.6)
Moist soil	O (n=6)	14.7 (2.4)	23.6 (15.2)	1.6 (1)	5.1 (0.2)	6.9 (1.7)	5.9 (1.9)
	Mineral (n=4)	15.2 (2.4)	2.4 (1)	0.1 (0)	4.2 (0.3)	3.8 (1.1)	3.8 (1.1)

Supplementary Table 4. Mean (SD) 2-pool decay coefficients for modelling microbial respiration post-burn. M_1 is the fractional active (or fast) C pool, and k_1 is the respiration rate constants for the fast C pool.

	Fast growth incubation		Affinity for post-fire environment incubation - Autoclaved		Affinity for post-fire environment incubation – Not autoclaved	
	k_1	M_1	k_1	M_1	k_1	M_1
Unburned soil	0.0036 (0.015)	0.096 (0.10)	0.045 (0.039)	0.095 (0.061)	0.014 (0.014)	0.28 (0.17)
Moist soil	0.16 (0.12)	0.019 (0.013)	0.046 (0.023)	0.096 (0.058)	0.069 (0.12)	0.14 (0.13)
Dry soil	0.016 (0.075)	0.034 (0.021)	0.062 (0.027)	0.072 (0.039)	0.058 (0.029)	0.074 (0.045)

Supplementary Table 5. Mean (SD) 2-pool decay coefficients for modelling microbial respiration post-burn. M_2 is the fractional slow C pool, and k_2 is the respiration rate constant for the slow C pool.

	Fast growth incubation		Affinity for post-fire environment incubation - Autoclaved		Affinity for post-fire environment incubation – Not autoclaved	
	k_2	M_2	k_2	M_2	k_2	M_2
Unburned soil	0.0025 (0.00098)	0.89 (0.0025)	0.00088 (0.00057)	0.89 (0.063)	0.00039 (0.00063)	0.71 (0.17)
Moist soil	0.0017 (0.00085)	0.97 (0.018)	0.00088 (0.00054)	0.87 (0.06)	0.00091 (0.00092)	0.84 (0.00091)
Dry soil	0.00098 (0.00072)	0.05 (0.024)	0.00053 (0.00025)	0.90 (0.064)	0.00052 (0.00031)	0.89 (0.093)

Supplementary Table 6. PERMANOVA results for full model for 16S from cDNA 24h after burning.

	Df	Sums of sqs	Mean sqs	F.Model	R^2	Pr(>F)	R^2 single-component model
Dominant Vegetation	2	0.000322	0.000161	2.0359	0.03604	0.001	0.03804
Pre-burn horizon thickness (cm)	1	0.000214	0.000214	2.6995	0.02389	0.002	0.02259
pH	1	0.000789	0.000788	9.9644	0.0882	0.001	0.13376
Total C (%)	1	0.000281	0.000281	3.5526	0.03145	0.001	0.0315
Total N (%)	1	0.00018	0.00018	2.277	0.04269	0.003	0.03408
Soil texture	4	0.000593	0.000148	1.8746	0.06637	0.001	0.08829
Burn treatment	2	0.000222	0.000111	1.3994	0.08195	0.05	0.07969
Soil horizon	1	0.69043	0.000167	2.1112	0.01869	0.011	0.04257
Residuals	78	0.006172	7.91E-05		0.69043		
Total	91	0.00894			1		

Supplementary Table 7. PERMANOVA results for full model from 16S gDNA 24h after burning

	Df	Sums of sqs	Mean sqs	F.Model	R^2	Pr(>F)	R^2 single-componentmodel
Dominant Vegetation	2	0.001197	0.000598	3.017	0.05246	0.001	0.04618
Pre-burn horizon thickness (cm)	1	0.000746	0.000746	3.7606	0.03269	0.001	0.04093
pH	1	0.001238	0.001238	6.2422	0.05427	0.001	0.04924
Total C (%)	1	0.000848	0.000848	4.2757	0.03717	0.001	0.05109
Total N (%)	1	0.00035	0.00035	1.7636	0.01533	0.032	0.05114
Soil texture	4	0.002038	0.000509	2.568	0.0893	0.001	0.11346
Burn treatment	2	0.000666	0.000333	1.6781	0.02918	0.017	0.04097
Soil horizon	1	0.00046	0.00046	2.3204	0.02017	0.002	0.04873
Residuals	77	0.015275	0.000198		0.66942		
Total	90	0.022818			1		

Supplementary Table 8. PERMANOVA results for full model for microbial community composition at end of 5-week fast-growth incubation.

	Df	Sums Of Sqs	Mean Sqs	F.Model	R^2	Pr(>F)	Individual model (R^2)
Dominant Vegetation	2	0.000578	0.000289	2.0566	0.03493	0.001	0.03434
Pre-burn horizon thickness (cm)	1	0.000372	0.000372	2.6458	0.02247	0.002	0.02668
pH	1	0.001791	0.001791	12.7459	0.10823	0.001	0.11184
Total C (%)	1	0.000657	0.000657	4.6747	0.03969	0.001	0.04664
Total N (%)	1	0.000206	0.000206	1.4633	0.01243	0.071	0.04832
Soil texture	4	0.001023	0.000256	1.8206	0.06184	0.001	0.08092
Burn treatment	2	0.000693	0.000346	2.4648	0.04186	0.001	0.09525
Soil horizon	1	0.000269	0.000269	1.9115	0.01623	0.011	0.05924
Residuals	78	0.010957	0.00014		0.66233		
Total	91	0.016543			1		

Supplementary Table 9. PERMANOVA results for full model for microbial community composition at end of 6-month post-fire affinity incubation.

	Df	Sums Of Sqs	Mean	Sqs F.Model	R^2	Pr(>F)	Individual model (R^2)
Dominant Vegetation	2	0.001674	0.000837	3.845	0.03293	0.001	0.03286
Pre-burn horizon thickness (cm)	1	0.00088	0.00088	4.0411	0.0173	0.001	0.02287
pH	1	0.003791	0.003791	17.4128	0.07456	0.001	0.08042
Total C (%)	1	0.001445	0.001445	6.6355	0.02841	0.001	0.03597
Total N (%)	1	0.000711	0.000711	3.2656	0.01398	0.001	0.03738
Soil texture	4	0.002902	0.000726	3.3324	0.05707	0.001	0.07111
Burn treatment	2	0.000806	0.000403	1.8507	0.01585	0.001	0.04714
Soil horizon	1	0.000689	0.000689	3.1666	0.01356	0.001	0.05143
Autoclave	1	0.000938	0.000938	4.3073	0.01844	0.001	0.01831
Residuals	170	0.03701	0.000218		0.7279		
Total	184	0.050845			1		

Supplementary Table 10. Observed OTUs in each laboratory sample*			
Burn treatment	Incubation treatment	Mean observed taxa (SD)	
		O horizon	A horizon
Control	24h post-burn (RNA)	689 (146)	767 (260)
	24h post-burn (DNA)	827 (210)	883 (205)
	5 weeks	881 (332)	1184 (420)
	6 months autoclaved + inoculated	614 (185)	685 (183)
Moist	24h post-burn (RNA)	680 (237)	630 (97)
	24h post-burn (DNA)	822 (221)	678 (99)
	5 weeks	904 (367)	988 (332)
	6 months autoclaved + inoculated	630 (176)	660 (208)
Dry	24h post-burn (RNA)	696 (334)	728 (235)
	24h post-burn (DNA)	1224 (576)	862 (298)
	5 weeks	870 (760)	740 (404)
	6 months autoclaved + inoculated	796 (440)	631 (194)

* Note – we present these data only to give a general picture of community composition across treatments. Observed OTU counts derived from sequencing data should not be interpreted as statistically robust estimates of richness.

Supplementary Table 11. All responding OTUs. See Johnson_Supplementary_Table_11 tab in supplementary-tables-11-and-16.xlsx

Supplementary Table 12. Number of OTUs from each phylum identified as responding taxa

Phylum	Number of survivor OTUs	Number of fast grower OTUs	Number of post-fire affinity OTUs
Acidobacteriota	3	2	
Actinobacteriota	4	13	6
Bacteroidota	1	3	
Bdellovibrionota		2	1
Chloroflexi	1		1
Firmicutes	6	13	4
Gemmatimonadota		1	2
Myxococcota			1
Planctomycetota	2	7	6

Proteobacteria	3	29	10
Verrucomicrobiota		1	

Supplementary Table 13. Number of OTUs from each class identified as responding taxa.

Class	Number of survivor OTUs	Number of fast grower OTUs	Number of post-fire affinity OTUs
Acidomicrobiia	3		2
Actinobacteria	3	11	3
AD3	1		1
Alphaproteobacteria	3	19	7
Bacilli	5	9	4
Bacteroidia	1	3	
Bdellovibrionia		1	1
Blastocatellia		2	
Clostridia	1	1	
Gammaproteobacteria		10	3
Gemmatimonadetes		1	2
Myxococcia			1
Oligoflexia		1	
Phycisphaerae		2	1
Planctomycetes	2	5	5
Symbiobacteriia		3	
Thermoleophilia	1	2	1
Verrucomicrobiae		1	

Supplementary Table 14. Mean whole-core fractional dry mass loss during burning grouped by dominant vegetation type and burn treatment. Standard deviation in parentheses.

**Dominant vegetation**	**Burn treatment**	**Fractional dry mass loss**
Picea spp.	dry	0.2 ± (0.19)
Picea spp.	moist	0.14 ± (0.07)
Pinus banksiana	dry	0.09 ± (0.05)
Pinus banksiana	moist	0.06 ± (0.03)
Populus tremuloides	dry	0.23 ± (0.21)

Populus tremuloides	moist	0.1 ± (0.03)

Supplementary Table 15. Primers used in this study [1]

Primer, 16S Illumina	Sequence (Illumina adaptor, *Barcode*, **Pad and linker,** Primer)
515f	AATGATACGGCGACCACCGAGATCTACAC*XXXXXXXX***TATGGTAATTGT**GTGYCAGCMGCCGCGGTAA
806r	CAAGCAGAAGACGGCATACGAGAT*XXXXXXXX***AGTCAGCCAGCC**GGACTACNVGGGTWTCTAAT
Read 1 seq	TATGGTAATTGTGTGYCAGCMGCCGCGGTAA
Read 2 seq	AGTCAGCCAGCCGGACTACNVGGGTWTCTAAT
Barcode seq	ATTAGAWACCCBNGTAGTCCGGCTGGCTGACT

Supplementary Table 16. Full PCR primers with barcodes. See Johnson_Supplementary_Table_16 tab in supplementary-tables-11-and-16.xlsx

Supplementary materials and methods

Study region details: Mean temperatures range from 13 °C in the summer to -17.5 °C in the winter, and annual precipitation is 300-400 mm [2]. Most precipitation falls in the summer (June-September) months. Precipitation during the summer averages 50 mm per month but can range from as little as no precipitation during droughts to >140 mm per month [3]. For example, in Hay River, located approximately 35 km north of WBNP, summer precipitation in 2014 dropped to 27 mm per month from a summer monthly average of 41 mm and in September 2014, no precipitation was recorded. In Fort Smith, located on the eastern edge of WBNP, monthly precipitation < 3 mm was recorded in June 2007, August 2009, July 2010, and September 2011 [3].

Site details: Sites were located between 0.1 and 1 km from roads and > 0.5 km from other sampling sites. We used a Garmin GPSMAP 64 GPS finder to reach each designated location. Upon arriving, we confirmed the dominant tree species and recorded slope and aspect. Samples were collected across a 2 x 2-meter grid. A collapsible PVC pipe square was used to map out the sampling grid. After sampling most field sites, we produced a second selection of random points in a more limited region with field-validated tree species dominance. This second random sample was designed to address identified gaps in species dominance in the initial sample that were the result of errors and limitations in the map products used, resulting in a total of 19 sites, with 6-7 sites under each dominant vegetation type. At each site, ten soil cores (15.24 cm x 7.62 cm dia.) were collected using a soil core sampler with clear plastic core liners and plastic end caps (Product IDs 405.09 and 418.09; AMS, American Falls, ID, USA) every 1 m within (and two at the center of) a 2 m x 2 m grid. Three of these cores were selected for the burn treatments (unburned, moist, dry) for each site.

Fire simulation: This heat flux was chosen to represent typical fire front propagation [4] and simulate a mid- to high-range crown fire [5, 6]. All the dry soil cores experienced some degree of combustion. Some but not all the moist soil cores ignited during the burn treatment.

pH: Briefly, we mixed mineral and organic soil with a 0.1 M $CaCl_2$ solution in 1:1 and 1:5 soil:solution ratios, respectively. Samples were incubated at room temperature and vortexed every 20 minutes for 1 hour. Samples were centrifuged at 10,000 x g for 1 minute, and pH of the supernatant was measured using a pH electrode (Orion Star A215, Thermo Fisher Scientific, MA, USA) [7].

Total C and N: Medium and low organic content soil and clay (Product IDs B2178, B2152, and B2184; EA Consumables, Pennsauken, NJ, USA) were used to calibrate the instrument (Flash EA 1112 CN Automatic Elemental Analyzer (Thermo Finnigan, Milan, Italy)). We excluded C and N values from site 10 control, moist burn soil, and dry burn soil for all relevant analyses due to an error at time of measurement.

KOH base trap: Base trap absorption of grams of CO_2 was calculated as the proportion (*P*) of the total base trap capacity for CO_2 absorption (*CCA*) that is used, multiplied by the trap capacity (modified from Strotmann et al. 2004). *P* is calculated as (Equation 2):

$$P = (EC_{init} - EC_{sample}) / (EC_{init} - EC_{sat}) \quad (2)$$

Where EC_{init}, EC_{sample}, EC_{sat} are the electrical conductivity of the absorbing OH ion solution initially (0.5 M KOH), at the time of measurement, and at full saturation (0.25 M K_2CO_3), respectively. The base trap capacity for absorption of g CO_2 is calculated as (Equation 3):

$$CCA = V \times C \times R \times M \quad (3)$$

Where *V* is the volume of the absorbing OH ion solution (0.015 L), *C* is the initial concentration of KOH, *R* is the mole to mole ratio of KOH present to CO_2 absorbed (0.5), and *M* is the molecular mass of CO_2 [8, 9].

Incubation setup: For both incubations, soil was packed to match the wet bulk density of the original core as recorded during the destructive sampling process post-burn treatment. Bulk densities ranged from 0.005-0.29 g cm^{-3} for the O horizon and 0.25-0.61 g cm^{-3} for the mineral soil. Finally, deionized water was added to each sample to bring all samples to equivalent soil moisture across all incubations from a given site. Target soil moistures were based on a water holding capacity of 175% g H_2O per g dry soil for O horizons and 55% water filled pore space for mineral soil. Seven of the 19 sites contained O horizons that we classified as Fibric Histosols, in which case we used a target moisture of 285% g H_2O per g dry soil, which is consistent with peatland moistures [10].

RNA and DNA: RNA and genomic DNA (gDNA) co-extractions were performed for each soil horizon of each sample with a blank extraction (identical methods but with empty tubes) for every 8 samples using RNeasy PowerSoil Total RNA Kits and RNeasy PowerSoil DNA Elution Kits (QIAGEN, Germantown, MD, USA), respectively. In brief, 0.5 – 1 g of O horizon or 2 g of mineral soil was added to a PowerBead tube. A phenol/chloroform nucleic acid extraction was performed following manufacturer's instructions. Homogenization and lysis were done using a FastPrep-24™ 5G bead beating grinder and lysis system (MP Biomedicals, Irvine, CA, USA) at a frequency of 6.5 m s^{-1} for 45 s at room temperature.

Residual gDNA contamination was removed from the RNA extracts using DNase Max Kits (QIAGEN, Germantown, MD, USA) following manufacturer's instructions. In brief, 40 µL of sample was combined with a DNase enzyme, buffer, and nuclease-free water and incubated at 37 °C for 20 min. The sample was then combined with 5 µL of DNase removal resin, incubated for 10 mins on a vortex adapter set to low, and centrifuged at 13,000 x g for 1 min. Supernatant was collected in a clean tube for further analysis.

RNA reverse transcription was carried out using Invitrogen SuperScript IV VILO Master Mix (ThermoFisher Scientific, Waltham, MA, USA) following manufacturer's instructions. A control reaction using no reverse transcriptase enzyme was carried out on one of every three RNA extracts to ensure minimum contamination by gDNA in the RNA sample. A Quant-iT RiboGreen RNA Assay Kit (ThermoFisher Scientific, Waltham, MA, USA) was used to assess copy DNA (cDNA) concentration and gDNA contamination levels.

PCR: In brief, PCR mixes contained 1.25 µL 515f forward primer (10 µM), 1.25 µL 806r reverse primer (10 µM), 1 µL cDNA or gDNA template, 12.5 µL Q5 Hot Start High-Fidelity 2X Master mix (New England BioLabs INC., Ipswich, MA, USA), 1.25 µL Bovine Serum Albumin (BSA) (20 mg mL^{-1}) (VWR, Radnor, PA, USA), and 7.75 µL nuclease-free water, in a 96-well plate. The plate was sealed and placed on an Eppendorf Mastercycler nexus gradient thermal cycler (Hamburg, Germany). Reactions were run at 98 °C for 2 minutes + (98 °C for 30 seconds + 58 °C for 15 seconds + 72 °C for 10 seconds) x 30 cycles + 72 °C for

2 minutes and 4 °C hold. The quality of PCR amplicon triplicates was assessed using gel electrophoresis, and triplicates were then pooled, purified, and normalized using SequalPrep Normalization Plate Kits (96-well) (ThermoFisher Scientific, Waltham, MA, USA). Samples were then pooled, and library cleanup was performed using a Wizard SV Gel and PCR Clean-Up System (Promega, Madison, WI, USA). The pooled library, including blanks, was submitted to the UW-Madison Biotechnology Center (Madison, WI, USA) for 2x250 paired end Illumina MiSeq sequencing. The library was sequenced twice using identical protocols to improve sequencing depth. Reads from the two sequencing runs were pooled by sample after sequence processing and before analysis.

Sequence processing: We quality filtered, trimmed (left trim 13 for both reads; truncation length 182 for forward reads, 161 for reverse reads), and dereplicated, learned errors (1M reads, randomized), picked operational taxonomic units (OTUs), and removed chimeras (consensus method) using dada2 [11] as implemented in Quantitative Insights Into Microbial Ecology (QIIME2) [12]. A total of 18,830,558 reads were obtained from amplicon sequencing. 6,360,490 reads were removed during the filtering processing, and taxonomy was assigned to the remaining 12,470,068 reads (24,940 mean 16S reads per sample) using the naïve Bayes classifier [13] in QIIME2 with the aligned 515f-806r region of the 99% OTUs from the SILVA database (SIVLA 138 SSU) [14–16]. We excluded one cDNA and two gDNA samples from further analysis due to low 16S reads per sample (<1000).

Statistics: We compared community composition across samples using weighted UniFrac dissimilarities on RNA- and DNA-based relative abundances and tested for significant effects of horizon (O horizon versus mineral soil), dominant vegetation, pre-burn horizon thickness, pH, total C and N, soil texture, and degree hours (DH) using a permutational multivariate ANOVA (PERMANOVA using the *adonis* function in *vegan* [17]) and used the *betadisp* function to test if variables differed in their dispersion. We used single-component models to compare the R^2 for each factor.

We used the rrnDB RDP Classifier tool (version 2.12) to obtain a mean 16S rRNA gene copy number for each genus in the fast growth dataset [18]. Briefly, for taxa with and without a genus-level assignment, we used the genus mean copy number or the mean copy number for all other taxa in this study, respectively, in the RDP database as the predicted copy number. We normalized OTU counts by dividing by the predicted copy number and summed the product of the predicted copy number and each OTU relative abundance for all OTUs in each sample to calculate the community weighted mean rRNA gene copy number.

References

1. Kozich JJ, Westcott SL, Baxter NT, Highlander SK, Schloss PD. Development of a dual-index sequencing strategy and curation pipeline for analyzing amplicon sequence data on the MiSeq Illumina sequencing platform. *Appl Environ Microbiol* 2013; **79**: 5112–5120.

2. ESWG. A national ecological framework for Canada. *Agriculture and Agri-Food Canada, Research Branch, Centre for Land and Biological Resources Research and Environment Canada, State of the Environment Directorate, Ecozone Analysis Branch*. 1995. Ottawa, Ontario/Hull, Quebec, Canada.

3. Environment and Climate Change Canada. "Historical data: Monthly Data report for 2014, Fort Smith A Northwest Territories." *Government of Canada*. June 1, 2021. https://climate.weather.gc.ca/historical_data/search_historic_data_e.html

4. Silvani X, Morandini F. Fire spread experiments in the field: Temperature and heat fluxes measurements. *Fire Saf J* 2009; **44**: 279–285.

5. Thompson DK, Wotton BM, Waddington JM. Estimating the heat transfer to an organic soil surface during crown fire. *Int J Wildl Fire* 2015; **24**: 120–129.

6. Frankman D, Webb BW, Butler BW, Jimenez D, Forthofer JM, Sopko P, et al. Measurements of convective and radiative heating in wildland fires. *Int J Wildl Fire* 2012; **22**: 157–167.

7. Braus MJ, Whitman TL. Standard and non-standard measurements of acidity and the bacterial ecology of northern temperate mineral soils. *Soil Biol Biochem* 2021; **160**: 108323.

8. Strotmann U, Reuschenbach P, Schwarz H, Pagga U. Development and evaluation of an online CO_2 evolution test and a multicomponent biodegradation test system. *Appl Environ Microbiol* 2004; **70**: 4621–4628.

9. Anderson JPE. Soil Respiration. In: Page AL (ed). *Methods of Soil Analysis: Part 2 Chemical and Microbiological Properties,* Second. 1983. American Society of Agronomy, pp 331–371.

10. Rydin H, Jeglum JK. Peatland Hydrology. *The Biology of Peatlands,* Second Edi. 2013. Oxford University Press, pp 148–174.

11. Callahan BJ, McMurdie PJ, Rosen MJ, Han AW, Johnson AJA, Holmes SP. DADA2: High-resolution sample inference from Illumina amplicon data. *Nat Methods* 2016; **13**: 581–583.

12. Bolyen E, Rideout JR, Dillon MR, Bokulich NA, Abnet CC, Al-Ghalith GA, et al. Reproducible, interactive, scalable and extensible microbiome data science using QIIME 2. *Nat Biotechnol* 2019; **37**: 852–857.

13. Bokulich NA, Kaehler BD, Rideout JR, Dillon M, Bolyen E, Knight R, et al. Optimizing taxonomic classification of marker-gene amplicon sequences with QIIME 2's q2-feature-classifier plugin. *Microbiome* 2018; **6**: 90.

14. Quast C, Pruesse E, Yilmaz P, Gerken J, Schweer T, Yarza P, et al. The SILVA ribosomal RNA gene database project: improved data processing and web-based tools. *Nucleic Acids Res* 2013; **41**: D590–D596.

15. Glöckner FO, Yilmaz P, Quast C, Gerken J, Beccati A, Ciuprina A, et al. 25 years of serving the community with ribosomal RNA gene reference databases and tools. *J Biotechnol* 2017; **261**: 169–176.

16. Yilmaz P, Parfrey LW, Yarza P, Gerken J, Pruesse E, Quast C, et al. The SILVA and "All-species Living Tree Project (LTP)" taxonomic frameworks. *Nucleic Acids Res* 2014; **42**: D643–D648.

17. Oksanen J, Blanchet FG, Friendly M, Kindt R, Legendre P, McGlinn D, et al. vegan: Community Ecology Package. *R Packag version 25-7*. 2020.

18. Stoddard SF, Smith BJ, Hein R, Roller BRK, Schmidt TM. rrnDB: Improved tools for interpreting rRNA gene abundance in bacteria and archaea and a new foundation for future development. *Nucleic Acids Res* 2015; **43**: D593–D598.
