# Supplementary material for: Experimentally determined traits shape bacterial community composition one and five years following wildfire
Source: Nat Ecol Evol. 2023 Jul 31;7(9):1419–31. doi: 10.1038/s41559-023-02135-4 (PMC10482699; doi:10.1038/s41559-023-02135-4)
Supplement: Supplementary file 2 — Reporting Summary [file 41559_2023_2135_MOESM2_ESM.pdf]

nature portfolio

Corresponding author(s): Thea Whitman

Last updated by author(s): Dec 14, 2022

Reporting Summary

Nature Portfolio wishes to improve the reproducibility of the work that we publish. This form provides structure for consistency and transparency in reporting. For further information on Nature Portfolio policies, see our Editorial Policies and the Editorial Policy Checklist.

Statistics

For all statistical analyses, confirm that the following items are present in the figure legend, table legend, main text, or Methods section.

n/a	Confirmed	
☐	☒	The exact sample size (*n*) for each experimental group/condition, given as a discrete number and unit of measurement
☐	☒	A statement on whether measurements were taken from distinct samples or whether the same sample was measured repeatedly
☐	☒	The statistical test(s) used AND whether they are one- or two-sided<br>*Only common tests should be described solely by name; describe more complex techniques in the Methods section.*
☐	☒	A description of all covariates tested
☐	☒	A description of any assumptions or corrections, such as tests of normality and adjustment for multiple comparisons
☐	☒	A full description of the statistical parameters including central tendency (e.g. means) or other basic estimates (e.g. regression coefficient) AND variation (e.g. standard deviation) or associated estimates of uncertainty (e.g. confidence intervals)
☐	☒	For null hypothesis testing, the test statistic (e.g. *F*, *t*, *r*) with confidence intervals, effect sizes, degrees of freedom and *P* value noted<br>*Give P values as exact values whenever suitable.*
☒	☐	For Bayesian analysis, information on the choice of priors and Markov chain Monte Carlo settings
☒	☐	For hierarchical and complex designs, identification of the appropriate level for tests and full reporting of outcomes
☒	☐	Estimates of effect sizes (e.g. Cohen's *d*, Pearson's *r*), indicating how they were calculated

Our web collection on statistics for biologists contains articles on many of the points above.

Software and code

Policy information about availability of computer code

Data collection	All data were collected in the lab, not using software or code.
Data analysis	All analyses in this paper were conducted in R, as detailed in the methods section. Code for the analyses conducted in the current study are available at https://github.com/DanaBJohnson/WoodBuffalo2019.

For manuscripts utilizing custom algorithms or software that are central to the research but not yet described in published literature, software must be made available to editors and reviewers. We strongly encourage code deposition in a community repository (e.g. GitHub). See the Nature Portfolio guidelines for submitting code & software for further information.

Data

Policy information about availability of data

All manuscripts must include a data availability statement. This statement should provide the following information, where applicable:

- Accession codes, unique identifiers, or web links for publicly available datasets
- A description of any restrictions on data availability
- For clinical datasets or third party data, please ensure that the statement adheres to our policy

The sequencing datasets generated during the current study are available in the NCBI SRA under bioproject number PRJNA913093. Field sequencing data are available at PRJNA564811 (2015 data) and PRJNA825513 (2019 data, available 31 December 2022). Non-sequencing data are deposited in the DOE ESS-DIVE repository under: https://data.ess-dive.lbl.gov/datasets/ess-dive-e00cfbe3d89f2c1-20220421T224630867

Human research participants

Policy information about studies involving human research participants and Sex and Gender in Research.

Reporting on sex and gender	NA
Population characteristics	NA
Recruitment	NA
Ethics oversight	NA

Note that full information on the approval of the study protocol must also be provided in the manuscript.

Field-specific reporting

Please select the one below that is the best fit for your research. If you are not sure, read the appropriate sections before making your selection.

☐ Life sciences ☐ Behavioural & social sciences ☒ Ecological, evolutionary & environmental sciences

For a reference copy of the document with all sections, see nature.com/documents/nr-reporting-summary-flat.pdf

Ecological, evolutionary & environmental sciences study design

All studies must disclose on these points even when the disclosure is negative.

Study description

Our overarching goal is to develop a fire ecology framework for soil microbes. Specifically, we aimed to identify bacteria with fire-related traits (fire survival, fast growth, and affinity for the post-fire environment), and (1) assess how the abundance of taxa with these different traits vary one and five years post-fire, and with increasing burn severity (2) determine whether there were trade-offs between these traits, and (3) determine how fire-induced changes to soil microbes and soil properties affect post-fire C cycling.

To this end, we collected soil cores from 19 sites. For each site, 3 fire treatments were applied to an individual core - control, moist soil burn, dry soil burn (i.e., N=19 for each burn treatment). Each site x fire treatment was then included in each of three experiments designed to identify fire-responsive traits (survival, fast growth, and affinity for the post-fire environment). Subsequent analyses were performed on organic and mineral horizons separately. Since some sites had no mineral horizon within the sampled depth (particularly in the Picea sp.-dominated sites), N=19 for organic horizons and N=12 for mineral horizons.

Research sample

The samples are soils collected from 19 sites across Wood Buffalo National Park in the Northwest Territories and Alberta, Canada. Samples were collected from 7 Picea sp.-dominated sites, 6 Pinus banksiana-dominated sites, and 6 Populus tremuloides-dominated sites that had not burned in the last 30 years. These sites were chosen using stratified random sampling using the Canadian National Fire Database, dominant vegetation from the Canadian National Forest Inventory, and soil type from FAO soil survey data, with the goal of representing the dominant vegetation and soil types of the southern half of the boreal and taiga plains ecoregions of northwestern Canada.

The study draws on bacterial community sequencing data from T Whitman, J Woolet, M Sikora, DB Johnson, E Whitman. 2022. Resilience in soil bacterial communities of the boreal forest from one to five years after wildfire across a severity gradient. Soil Biology and Biochemistry 172, 108755, which are available in the NCBI SRA at PRJNA564811 (2015 data) and PRJNA825513 .

Sampling strategy

Sites that had not burned in the previous 30 years or more were selected using stratified random sampling using the Canadian National Fire Database, dominant vegetation from the Canadian National Forest Inventory, and soil type from FAO soil survey data. Sites were located between 0.1 and 1 km from roads and > 0.5 km from other sampling sites. We used a Garmin GPSMAP 64 GPS finder to reach each designated location. Upon arriving, we confirmed the dominant tree species and recorded slope and aspect. Samples were collected across a 2 x 2-meter grid. A collapsible PVC pipe square was used to map out the sampling grid. After sampling most field sites, we produced a second selection of random points in a more limited region with field-validated tree species dominance. This second random sample was designed to address identified gaps in species dominance in the initial sample that were the result of errors and limitations in the map products used, resulting in a total of 19 sites, with 6-7 sites under each dominant vegetation type. At each site, ten soil cores (15.24 cm x 7.62 cm dia.) were collected using a soil core sampler with clear plastic core liners and plastic end caps (Product IDs 405.09 and 418.09; AMS, American Falls, ID, USA) every 1 m within (and two at the center of) a 2 m x 2 m grid.

Sample size and sampling strategy were chosen to maximize representation of the region, using the stratified random sampling approach described above. This sample number was sufficient to achieve our goal for this sampling design, which was to identify bacterial taxa with fire-responsive traits in this region: in the lab-burned dry cores, these taxa represented a mean of 32% of the total reads. With a higher sample size, we might have been able to conclusively identify lower-abundance or rarer taxa that have these traits. However, given these taxa tend to be lower-abundance and rarer across samples in the first place, we expect that adding them to our list of trait-identified taxa would have relatively small effects on the main trends illustrated in Figures 4-6, and also means our approach is generally conservative. For soil properties, we were able to detect significant changes in C, C:N, and pH, using standard statistical tests (ANOVA, Wilcoxon signed rank test), indicating our sample size was sufficiently large to assess these changes. Where

	we did not detect significant differences (e.g., total N%), the effect sizes were generally relatively small, and perhaps of limited ecological relevance. They also are explainable - for example, during combustion of OM, C losses begin around 100 °C, whereas N volatilization begins around 200 °C, so it makes sense that we detected significant differences in %C but not %N.
Data collection	Soil texture was determined using a physical analysis hydrometer at the UW-Madison Soil and Forage Lab. Soil pH was measured by DBJ as detailed in Supplementary. Soil subsamples were prepped for total C and N analysis by DBJ and analyzed as detailed in Methods. Soil respiration was measured by DBJ as detailed in Supplementary. RNA and DNA concentrations were measured by DBJ as detailed in Methods and Supplementary. RNA and DNA extraction was carried out by DBJ and sequencing was done by the UW-Madison Biotechnology Center as described in Methods and Supplementary.
Timing and spatial scale	We collected 10 soil cores each from 19 sites within Wood Buffalo National Park in the Northwest Territories and Alberta, Canada between June 13 and 17, 2019. One to seven sites per day were sampled and each site was only sampled once in an effort to collect all samples within a relatively short time period to avoid confounding effects of seasonality. Exact latitude and longitude of each site are given in Supplemental Table S1.
Data exclusions	C and N values from site 10 control, moist burn soil, and dry burn soil were excluded for all relevant analyses due to an error at time of measurement. We excluded one cDNA and two gDNA samples from further analysis due to low 16S reads per sample (<1000).
Reproducibility	We have not yet attempted to reproduce the experiment in its entirety, from core collection to final analyses. However, we intentionally designed our approach to be as replicable as possible. Specific elements to improve replicability include the following: Allowing cores to air dry to moisture contents typical of drought in the region of study before imposing moisture and burn treatments means these treatments could be repeated with high replicability. Using a cone calorimeter to deliver specific heat fluxes, representative of a crown fire in this region, means the burn simulations are also highly replicable. Subsequent experiments and analyses draw on standard methods and protocols wherever possible, ensuring replicability is likely. Finally, all our data and analytical code are public, allowing for our analyses to be reproduced.
Randomization	Samples were not allocated into groups - every treatment was applied to every sample.
Blinding	During data collection and analysis, all samples were given a numeric identity that did not indicate their related treatment. We did not attempt to mask soil horizon identity because it is visually distinctive.

Did the study involve field work? ☒ Yes ☐ No

Field work, collection and transport

Field conditions	Temperature during sampling ranged from 10-30 °C and total precipitation over the 5 day sampling period was 8.4 mm.
Location	Exact sampling locations and site properties are given in detail in Supplemental Table S1.
Access & import/export	All samples were collected within the Wood Buffalo National Park under permit # WB-2019-31497
Disturbance	Fieldwork was designed to generally be low-impact, restricted to hiking by foot into the sites and collecting small soil cores, spaced at least 1 m from each other. We worked with the parks staff to ensure sensitive areas were not sampled.

Reporting for specific materials, systems and methods

We require information from authors about some types of materials, experimental systems and methods used in many studies. Here, indicate whether each material, system or method listed is relevant to your study. If you are not sure if a list item applies to your research, read the appropriate section before selecting a response.

Materials & experimental systems

n/a	Involved in the study	
☒	☐	Antibodies
☒	☐	Eukaryotic cell lines
☒	☐	Palaeontology and archaeology
☒	☐	Animals and other organisms
☒	☐	Clinical data
☒	☐	Dual use research of concern

Methods

n/a	Involved in the study	
☒	☐	ChIP-seq
☒	☐	Flow cytometry
☒	☐	MRI-based neuroimaging
